# Micro-RNA let-7a-5p Derived From Mesenchymal Stem Cell-Derived Extracellular Vesicles Promotes the Regrowth of Neurons in Spinal-Cord-Injured Rats by Targeting the HMGA2/SMAD2 Axis

**DOI:** 10.3389/fnmol.2022.850364

**Published:** 2022-03-25

**Authors:** Ying Wang, Tianyu Han, Ruocheng Guo, Peiwen Song, Yunlei Liu, Zuomeng Wu, Jichao Ai, Cailiang Shen

**Affiliations:** ^1^Department of Medical Imaging, The First Affiliated Hospital of Anhui Medical University, Hefei, China; ^2^Department of Orthopedics (Spinal Surgery), The First Affiliated Hospital of Anhui Medical University, Hefei, China; ^3^Department of Clinical Laboratory, No. 2 People’s Hospital of Fuyang, Fuyang, China; ^4^Department of Orthopedics, No. 2 People’s Hospital of Fuyang, Fuyang, China

**Keywords:** spinal cord injury (SCI), mesenchymal stem cells (MeSH ID D059630), extracellular vesicles (EVs), neural stem cells, SMAD 2

## Abstract

Spinal cord injury (SCI) often causes neuronal and axonal damage, resulting in permanent neurological impairments. Mesenchymal stem cells (MSCs) and extracellular vesicles (EVs) are promising treatments for SCI. However, the underlying mechanisms remain unclear. Herein, we demonstrated that EVs from bone marrow-derived MSCs promoted the differentiation of neural stem cells (NSCs) into the neurons and outgrowth of neurites that are extending into astrocytic scars in SCI rats. Further study found that let-7a-5p exerted a similar biological effect as MSC-EVs in regulating the differentiation of NSCs and leading to neurological improvement in SCI rats. Moreover, these MSC-EV-induced effects were attenuated by let-7a-5p inhibitors/antagomirs. When investigating the mechanism, bioinformatics predictions combined with western blot and RT-PCR analyses showed that both MSC-EVs and let-7a-5p were able to downregulate the expression of SMAD2 by inhibiting HMGA2. In conclusion, MSC-EV-secreted let-7a-5p promoted the regrowth of neurons and improved neurological recovery in SCI rats by targeting the HMGA2/SMAD2 axis.

## Introduction

Spinal cord injury (SCI) causes necrosis, demyelination of axons, and neuronal apoptosis, resulting in the disruption of the motor and sensory tracts of the spinal cord, which leads to severe neurological impairment (Assinck et al., 2017; O’Shea et al., 2017). The discovery of endogenous neural stem cells (NSCs) in the spinal cord has been a promising strategy for the treatment of SCI (Stenudd et al., 2015). However, further studies have shown that although these endogenous NSCs could be activated and then migrate into injured lesions after SCI, most of them differentiated into astrocytes forming glial scars due to the severe microenvironment following SCI. The over-formation of astrocytic scars haven been proved to be one of the reasons for the failure of the reorganization and regeneration of neural circuits. Therefore, it is critical to promote the differentiation of endogenous NSCs into neurons and oligodendrocytes.

Mesenchymal stem cells (MSCs) were first found in the bone marrow due to their self-renewing, multipotent abilities; they are obtained from various sources, and have been widely used for the treatment of SCI (Prockop et al., 2000; Cofano et al., 2019; Yamazaki et al., 2020). Initially, MSCs were transplanted to injured lesions in an attempt to differentiate them into oligodendrocytes and neurons, replacing the damaged nerve cells (Brazelton et al., 2000; Mezey et al., 2000). However, emerging studies have found that the beneficial biological effects of transplanted MSCs are mainly associated with their paracrine activity (Nakajima et al., 2012). Based on this evidence, conditioned medium (CM), which is composed of various factors secreted by MSCs, is widely used in the treatment of neurological disorders (Gnecchi and Melo, 2009; Cantinieaux et al., 2013). In addition, studies have reported that CM from MSCs can promote the regeneration of neruons and remyelination of axons by regulating the differentiation of endogenous NSCs, as well as protect these NSCs from apoptosis by generating a favorable environment against secondary injury following primary SCI (Fang et al., 2018; Niu et al., 2019; Song et al., 2020).

With further understanding of paracrine signaling from cells, extracellular vesicles (EVs) in MSC-CM were discovered (Chen et al., 2010; Keshtkar et al., 2018). EVs are membrane particles secreted by nearly all cell types and carry various bioactive cargos, including microRNAs (miRNAs) (Phinney et al., 2015). miRNAs are short RNA molecules, 20–22 nucleotides in length, that mainly work as negative regulators by degrading or repressing the targeted mRNAs in a variety of cellular processes (Vishnoi and Rani, 2017). Further studies have shown that miRNAs are critical regulators of stem cell biology, including proliferation and differentiation (Mens and Ghanbari, 2018; Vieira et al., 2018). Many miRNAs have been reported to be present in MSC-EVs (including the let-7 family, miRNA-21, miRNA-22, and miRNA-133) (Phinney and Pittenger, 2017). Moreover, some of these miRNAs secreted by MSCs play a crucial role in mediating axonal sparing, glial formation, and neural plasticity (Ghibaudi et al., 2017; Fang et al., 2021). However, whether these miRNAs could regulate the differentiation of endogenous NSCs, which are activated following SCI, and their molecular and cellular mechanisms remain unclear.

In this study, we focused on let-7a-5p, a member of the let-7 family, which contributes to axonal sparing, myelination, and neural regeneration (Kawahara et al., 2012). We demonstrated that both MSC-EVs and let-7a-5p are able to promote the differentiation of NSCs into neurons and that these MSC-EV-induced biological effects could be attenuated by the knockdown of let-7a-5p, indicating that MSC-EV-mediated differentiation of NSCs partly depends on let-7a-5p. Moreover, by detecting the expression of high-mobility group A 2 (HMGA2) and SMAD2, we found that let-7a-5p downregulated the TGF-beta/Smad signaling pathway by targeting HMGA2. *In vivo*, treatment with let-7a-5p and MSC-EVs promoted neurons and neurite outgrowth around the cavity in SCI rats. Moreover, the MSC-EVS-induced effects on neurite outgrowth and regulation of SMAD2 expression in injured lesions were suppressed by inhibiting let-7a-5p in SCI rats.

## Materials and Methods

### Mesenchymal Stem Cells Culture and the Collection of Mesenchymal Stem Cell-Extracellular Vesicles

The details of MSC culture have been described in our previous studies (Fang et al., 2018). MSCs were obtained from the bone marrow and cultured in low-glucose DMEM (Hyclone, United States) with 10% fetal bovine serum (FBS, Gibco, United States) in the presence of a 1% antibiotic solution. The cells were passaged when 90% confluence was reached. Passage 3 BMSCs were reseed and used to collect EVs. The supernatant was harvested when the BMSCs reached 90% confluence. EV collection was performed as described in our previous study (Han et al., 2021). Briefly, the harvested supernatant was centrifuged at 500 × *g* for 10 min, followed by centrifugation at 2,000 × *g* for 20 min and 10,000 × *g* for 45 min at 4°C to remove debris. Subsequently, the collected medium was re-centrifuged at 100,000 × *g* for 70 min at 4°C to obtain BMSC-EVs. Transmission electron microscopy, dynamic light scattering, and western blot analysis (CD 63, CD9, and TSG 101) were used to identify EVs (Supplementary Figure 1; Han et al., 2021). The EVSs were then dissolved in 100 μL of PBS and stored at −80°C.

### Neural Stem Cells Culture, Differentiation, and Transfection

Neural stem cells were cultured as described in our previous study (Fang et al., 2018). The cells were isolated from the subventricular zone and cultured as suspended neurospheres for 7 days in DMEM/F12 (Gibco, United States) containing 2% B27 (Gibco, United States), 20 ng/mL epidermal growth factor (EGF) (Gibco, United States), and 10 ng/mL basic fibroblast growth factor (bFGF) (Gibco, United States).

The overexpression or knockdown of let-7a-5p in NSCs was performed using 50 nM let-7a-5p mimics or inhibitors (Nanjing Kaien, China), non-target control miRNA mimics (NC mimics), or a scrambled control sequence (anti-NC) for NCs or anti-NCs. The HiPerfect transfection reagent (Hilden, Germany) was used following the manufacturer’s instructions. The sequences were as follows: let-7a-5p mimic, 5′-UGAGGUAGUAGGUUGUAUA GUU-3′; let-7a-5p inhibitor, 5′-AACUAUACAACCUACUAC CUCA-3′; NC mimic: 5′-UUCUCCGAACGUGUCACGUTT-3′; anti-NC: 5′-CAGUACUUUUGUGUAGUACAA-3′.

Passage 2 neurospheres or transfected neurospheres were dissociated by trypsin and cultured in 10% FBS-DMEM/F12 for 24 h. The medium was changed to DMEFM/F12 with or without BMSC-EVs. NSCs were cultured for 7 days and then used for immunofluorescence.

### RNA Extraction and Quantitative PCR

Total RNA of NSCs and 3 cm length of spinal cord tissues (center of the epicenter of the injured lesion) were extracted by using TRIzol (Gibco) following the manufacturer’s instructions, and cDNA was synthesized using the Superscript III RT Reaction Mix (Invitrogen). Quantitative PCR was performed using the SYBR Green master mix (Applied Biosystems) and a RealPlex2 Mastercycler (Eppendorf). mRNA expression was normalized to that of GAPDH and U6 was used to normalize miRNA expression. Sequences of transcript-specific primers were as followings: HMGA2 5′-GAGACCATTGGAGAAAAACGGC-3′, 5′-AATCTTCCTCTGCGGACTCTTGCG-3′; SMAD2 5′-CAGG AAGAGAAGTGGTGTGA-3′, 5′-ATACTGGAGGCAGAACTG GT-3′; GAPDH 5′-ACAGTCCATGCCATCACTGCC-3′, 5′-AC AGTCCATGCCATCACTGCC-3′; let-7a-5p 5′-CGGTGAGGTA GTAGGTTG-3′, 5′-GCAGGGTCCGAGGTATTC-3′; U6 5′-CT CGCTTCGGCAGCACA-3′, AACGCTTCACGAATTTGCGT.

### Animal Protocols

Details of the SCI procedure have been outlined in our previous study (Song et al., 2020). Briefly, laminectomy was performed at the T10 level in female Wistar rats. Rats were divided randomly into sham, SCI (control, treated with PBS), BMSC-EV-treated, let-7a-5p agomir-treated, and BMSC-EVs + let-7a-5p-antagomir-treated groups. A direct weight-drop injury to the spinal cord was induced *via* an infinite-horizon spinal cord impactor (IH-0400). A polyethylene catheter (PE-10, o.d. 0.61 mm) was placed at the injured level for intrathecal injection. The injured rats received a continuous injection of 25 μl PBS, 25 μl ml BMSC-EV-injection, 50 nmol let-7a-5p agomir (Ribobio, China), and 25 μl BMSC-EVs + 50 nmol let-7a-5p-antagomir (Ribobio, China) for 3 days. The Basso, Beattie, and Bresnahan (BBB) open-field test was used to investigate the motor function of the lower extremities of SCI rats blindly by two independent individuals at different time points (days 1, 4, 7, 14, 17, 21, 24, and 28) post-injury. The inclined plane test was also used to evaluate the neurological outcomes. It was performed on a testing apparatus and the maximum angle was recorded as the rat retained its position for more than 5 s without falling. Animal procedures were approved by the Ethics Committee of Anhui Medical University (No. 20191064) in accordance with the guidelines of the Declaration of Helsinki revised in Edinburgh in 2000.

### Tissue Processing and Immunofluorescence Staining

Spinal cord tissues were obtained from the rats 4-weeks post-injury and fixed in 4% paraformaldehyde. A 4-μm-thick longitudinal slice was obtained using a Leica RM2135 electric slicer. The following primary antibodies were used in the overnight incubation at 4°C: rabbit anti-Map-2 for neurons (1:500; Abcam, United Kingdom), mouse anti-glial fibrillary acidic protein (GFAP) for astroglia (1:1,000; Abcam, United Kingdom), and myelin basic protein (MBP) for oligodendrocytes (1:1,000; Abcam, United Kingdom) followed by incubation with primary antibodies for 1 h at room temperature: Alexa Fluor 488 (green, 1:50; Elabscience, China) and Cy3 (red, 1:50; Elabscience, China). The sections were observed and photographed using a DM-6B fluorescence microscope (Leica, Germany) connected to a computer screen. The percentage of positive areas was calculated using Image J.

### Dual-Luciferase Reporter Analysis

The HMGA2 3-UTR sequence containing the target gene of let-7a-5p or a mutant reporter of HMGA2 was cloned into a luciferase vector to construct the HMGA2-wt or HMGA2-mut (GenScript, China). NSCs were co-transfected with 50 nM of let-7a-5p mimics or NC mimics and HMGA2-wt or HMGA2-mut. After transfection for 48 h, luciferase activity was determined using the Dual-luciferase Reporter Assay System.

### Statistical Analysis

Data are presented as mean ± standard deviation, and statistical analyses were performed using SPSS software (version 16.0; Chicago, IL, United States). The data between two groups were analyzed using Student’s *t-*test to test the statistical significance. Statistical significance was set at a *p*-value of <0.05. For cell counting, 5–20 random fields containing a total of 500–1,000 cells were randomly selected. The number of positive cells was blindly quantified by two different individuals.

## Results

### BMSC-Extracellular Vesicles and let-7a-5p Promoted the Differentiation of Neural Stem Cells Into Neurons

**FIGURE 1:**
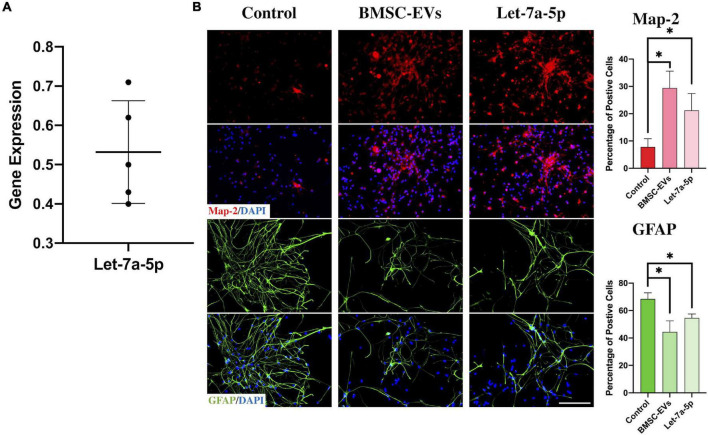
BMSC-EVs and let-7a-5p promoted the differentiation of NSCs into neurons. **(A)** PCR confirmed the presence of let-7a-5p in BMSC-EVs (*n* = 5). **(B)** The BMSC-EVs or let-7a-5p mimics were added to the NSCs and co-cultured for 7 days. The results showed that the percentage of Map-2 positive neurons was increased along with a reduction of GFAP positive astrocytes compared with the control groups by the addition of BMSC-EVs or let-7a-5p (*n* = 5; data are mean ± S.D; Student’s *t-*test was used for comparisons. *p < 0.05; scale bars, 100 μm).

As previously reported (Kim et al., 2017; Harrell et al., 2019), miRNAs are one of the components of EVs and play a key role in exerting their biological effects. To determine whether let-7a-5p was present in the BMSC-EVs, we first obtained EVs from bone marrow-derived MSCs and detected let-7a-5p expression by RT-PCR (Figure 1A). The results showed that let-7a-5p was present in all the samples from five individual rats. Next, to investigate the effects of BMSC-EVs and let-7a-5p on the differentiation of NSCs, NSCs were co-cultured with BMSC-EVs or let-7a-5p mimics for 7 days and the percentage of neurons and astrocytes was calculated by immunofluorescence. In the control group, approximately 70% of the cells were GFAP-positive astrocytes and 15% were microtubule-associated protein 2 (Map-2)-positive neurons (Figure 1B). In contrast, the addition of BMSC-EVs to NSCs increased the percentage of Map-2 positive neurons to 40%, and the proportion of GFAP-positive astrocytes decreased to 44% (Figure 1B). Similar to the biological effects of BMSC-EVs, the transfection of let-7a-5p mimics to NSCs increased the percentage of neurons to 27% with a reduction in the proportion of astrocytes compared with the control groups (Figure 1B). All these results indicated that in mediating the differentiation of NSCs, BMSC-EVs and let-7a-5p had similar biological functions.

**FIGURE 2:**
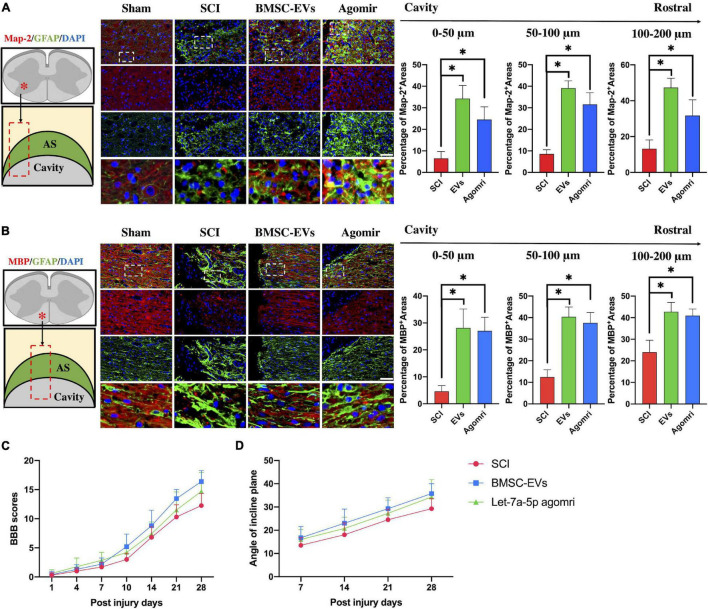
BMSC-EVs and let-7a-5p agomirs promoted the regeneration of neurons and their outgrowth in the glial scar surrounding the cavity, resulting in a better neurological outcome compared with the SCI rats. **(A,B)** In SCI rats, an astrocytic scar boundary was formed around the cavity, and Map-2 and MBP positive neurons were seldom present within the astrocytic scar. In contrast, the rats that received either BMSC-EVS or let-7a-5p agomir treatment had a significantly increased amounts of Map-2 and MBP positive neurons in the glial scars around the cavity (*n* = 5; data are mean ± S.D; Student’s *t-*test was used for comparisons. *p < 0.05; scar bar, 50 μm). **(C,D)** BBB scores and incline plane test showed that the neurological outcomes were improved in rats that received either BMSC-EVs or let-7a-5p agomirs compared with SCI rats (*n* = 10; data shown as mean ± S.D). (The term BMSC-EVs is abbreviated as EVs and let-7a-5p agomir is abbreviated as agomir).

In addition, we detected the histology of injured lesions and neurological function in rats injected with BMSC-EVs or let-7a-5p agomirs. The histological immunofluorescence results showed that in SCI rats, the glial scar boundary consisting of GFAP-positive astrocytes was formed around the cavity within few neurons at 4 weeks post-injury (Figures 2A,B). In contrast, in the glial scar boundary, the BMSC-EVs or let-7a-5p-agomir-treated rats had more Map-2-postive neurons in gray matters and MBP-positive myelin sheaths in white matter (Figures 2A,B). Moreover, neurite outgrowth of neurons and the axons extended into the scar and toward the cavity (Figures 2A,B). Consistent with the histology of the injured lesions, the BMSC-EVs or let-7a-5p-agomir-treated rats had better neurological outcomes than SCI rats (Figures 2C,D). All these results indicated that both BMSC-EVs and let-7a-5p were able to promote the regeneration of neurons and their neurite outgrowth, as well as inhibit the over-formation of astrocyte scars, resulting in a better neurological recovery following SCI.

### The Inhibition of let-7a-5p Attenuated the BMSC Extracellular Vesicle-Induced Effects *in vitro* and *in vivo*

**FIGURE 3:**
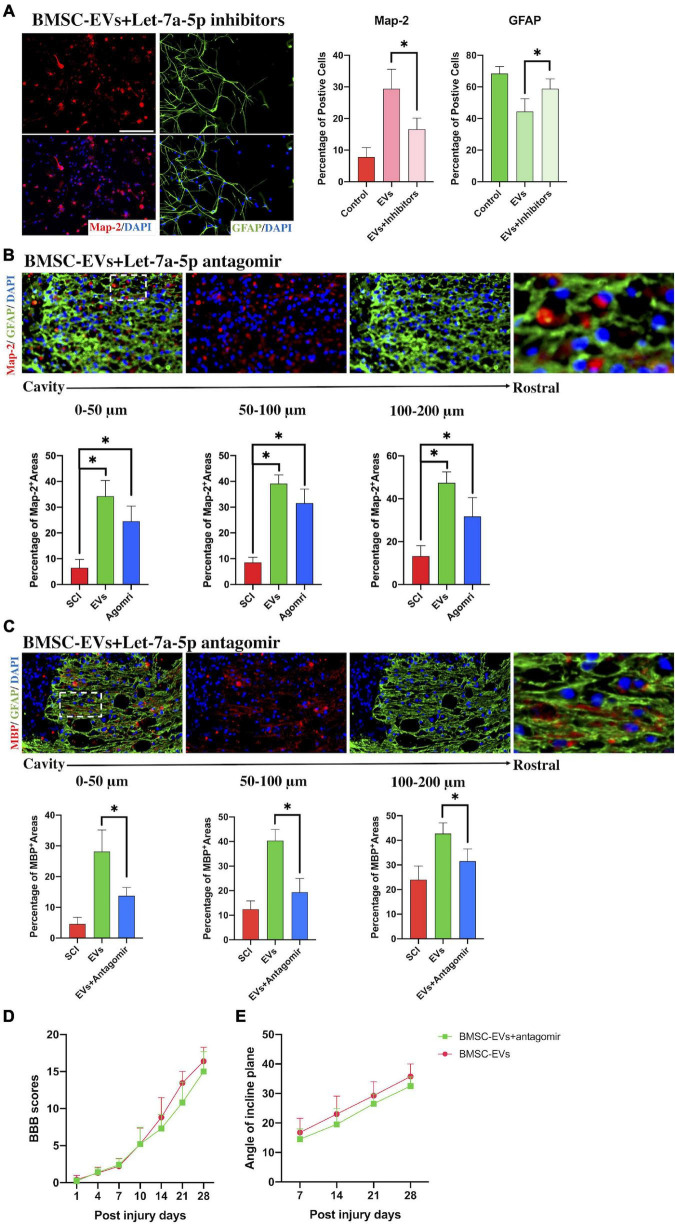
The inhibition of let-7a-5p attenuated the BMSC-EV-induced effects *in vitro* and *in vivo*. **(A)** The transfection of let-7a-5p inhibitors to NSCs in the presence of BMSC-EVs reduced the percentage of neurons and increased the proportion of astrocytes compared with the NSCs that only received the BMSC-EV treatment (*n* = 5; data are mean ± S.D; Student’s *t-*test was used for comparisons. *p < 0.05; scale bar, 100 μm). **(B,C)** The addition of let-7a-5p antagomirs reduced the Map-2 and MBP positive areas in glial scars in BMSC-EV-treated SCI rats (*n* = 5, data are mean ± S.D; Student’s *t-*test was used for comparisons. *p < 0.05; scar bar, 50 μm) and resulted in a worse BBB score **(D)** and lower max angle of incline plane tests **(E)** compared with the rats that only received BMSC-EV treatment (*n* = 10; data shown as mean ± S.D). The term BMSC-EVs is abbreviated as EVs, let-7a-5p agomir is abbreviated as agomir, and let-7a-5p inhibitors are abbreviated as inhibitors.

To determine whether qrKindly confirm whether the term [BMSC] is fine in line XXX.let-7a-5p secreted from BMSCs participated in mediating the differentiation of NSCs, let-7a-5p inhibitors were transfected into NSCs and co-cultured with BMSC-EVs for 7 days. The results of immunofluorescence revealed that compared with the BMSC-EV-treated NSCs, the let-7a-5p inhibitor-transfected NSCs had a lower percentage of Map-2-postive neurons and a higher proportion of GFAP-positive astrocytes following 7 days of co-culture with BMSC-EVs (Figure 3A), indicating that the BMSC-EV-induced biological effects on the differentiation of NSCs were partly repressed by the transfection of let-7a-5p inhibitors.

To further study whether the BMSC-EV-induced neurological improvement in SCI rats was associated with let-7a-5p, SCI rats were treated with the let-7a-5p antagomirs in the presence of BMSC-EVs. Compared with the BMSC-EV-treated SCI rats, the SCI rats that received both BMSC-EVs and antagomirs had fewer neurons and axons in the astrocytic scar around the cavity (Figures 3B,C), resulting in a decrease in BBB scores and the max angle of incline plane tests (Figures 3D,E). This indicated that the BMSC-EV-induced improvement was partly associated with let-7a-5p within EVs.

### By Targeting HMGA2, let-7a-5p Inhibited SMAD2 Expression

To further study the mechanism by which let-7a-5p mediates the differentiation of NSCs, the TargetScan database was used to predict the downstream molecular genes. It showed that a binding site of let-7a-5p was found at position 21–28 of the high mobility group protein A2 (HMGA2) 3′UTR. HMGA2 has been reported to play important roles in cell proliferation, differentiation, and migration by activating transforming growth factor beta (TGF-β)/Smad signaling (Dutta et al., 2017; Zhou et al., 2020). The TGF-beta/Smad signaling pathway could be significantly inhibited by the silencing of HMGA2, resulting in the alteration of apoptosis, migration, and invasion of cancer (Shi et al., 2016a,b). In addition, TGF-β has also been shown to be upregulated in injured lesions following SCI and is closely associated with the formation of glial scars (Buss et al., 2008; Zhang, 2009; Song et al., 2019). Therefore, we hypothesized that let-7a-5p mediates the differentiation of NSCs by targeting HMGA2, which represses TGF-beta/Smad signaling.

**FIGURE 4:**
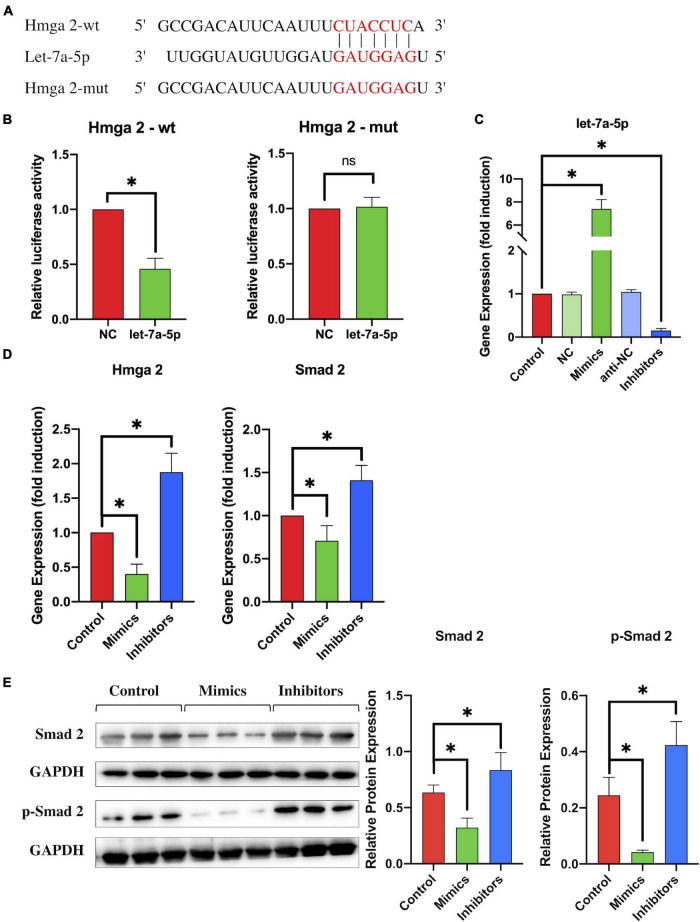
By targeting HMGA2, let-7a-5p downregulated the expression of SMAD2. **(A)** The target sequence for let-7a-5p in the 3′-UTR of HMGA2 and the mutated target sequence. **(B)** HMGA2-wt or HMGA2-mut was transfected into NSCs together with let-7a-5p mimics or NC mimics. Dual luciferase reporter analysis confirmed the direct recognition of the HMGA2 3′-UTR by let-7f-5p (*n* = 3; data are mean ± S.D; Student’s *t-*test was used for comparisons; *p < 0.05, n.s. *p* > 0.05). **(C)** NSCs were transfected with NC mimics, let-7a-5p mimics, anti-NCs, or let-7a-5p inhibitors respectively; RT-PCR analysis was used to detect the expression of let-7a-5p in NSCs. The analysis showed that transfection of let-7a-5p mimics was able to increase the expression of let-7a-5p. In contrast, the expression of let-7a-5p was reduced by the transfection of let-7a-5p inhibitors after 3 days of NSC culturing (*n* = 3). **(D)** RT-PCR detected the expression of HMGA2 and SMAD2 in NSCs with the addition of let-7a-5p mimics or inhibitors (*n* = 3). **(E)** Western-blot analysis showed that the SMAD2 and p-SMAD2 expression was repressed by the transfection of let-7a-5p mimics. In contrast, the transfection of let-7a-5p increased SMAD2 and p-SMAD2 expression after 3 days of NSC culture (*n* = 3; data are mean ± S.D; Student’s *t-*test was used for comparisons., gene expressions were normalized to the GAPDH (for mRNA) and U6 snRNA (for miRNA), *p < 0.05, ns *p* > 0.05. The term let-7a-5p mimics is abbreviated as mimics and let-7a-5p inhibitors is abbreviated as inhibitors).

To test this hypothesis, we first used dual-luciferase reporter assays to identify the correlation between HMGA2 and let-7a-5p (Figure 4A). Co-transfection of HMGA 3UTR-wt and let-7a-5p mimics resulted in reduction in luciferase activity (Figure 4B), indicating that let-7a-5p mimics targeted the 3′UTR binding site of HMGA2. Next, to investigate whether let-7a-5p was able to affect the expression of HMGA2 in NSCs, let-7a-5p mimics/inhibitors were transfected into NSCs. PCR analysis revealed that the expression of let-7a-5p in NSCs increased/decreased according to transfection of let-7a-5p mimics/inhibitors compared with the NC group (Figure 4C). Subsequently, the expression of HMGA2 and SMAD2 in NSCs was detected after transfection with let-7a-5p mimics or inhibitors. As expect, the transfection of let-7a-5p markedly reduced the expression of HMGA2 and SMAD2 in NSCs (Figure 4D). In contrast, HMGA2 and SMAD2 expression was significantly increased by transfection with the let-7a-5p inhibitor. In addition, we used western blotting to detect the expression of SMAD 2 and p-SMAD2, revealing that the transfection of let-7a-5p mimics to NSCs significantly reduced both SMAD2 and p-SMAD2 expression, whereas let-7a-5p inhibitor transfection increased the expression of SMAD2 and p-SMAD2 (Figure 4E). All these results indicated that let-7a-5p was able to downregulate the expression of SMAD2 by targeting HMGA2.

**FIGURE 5:**
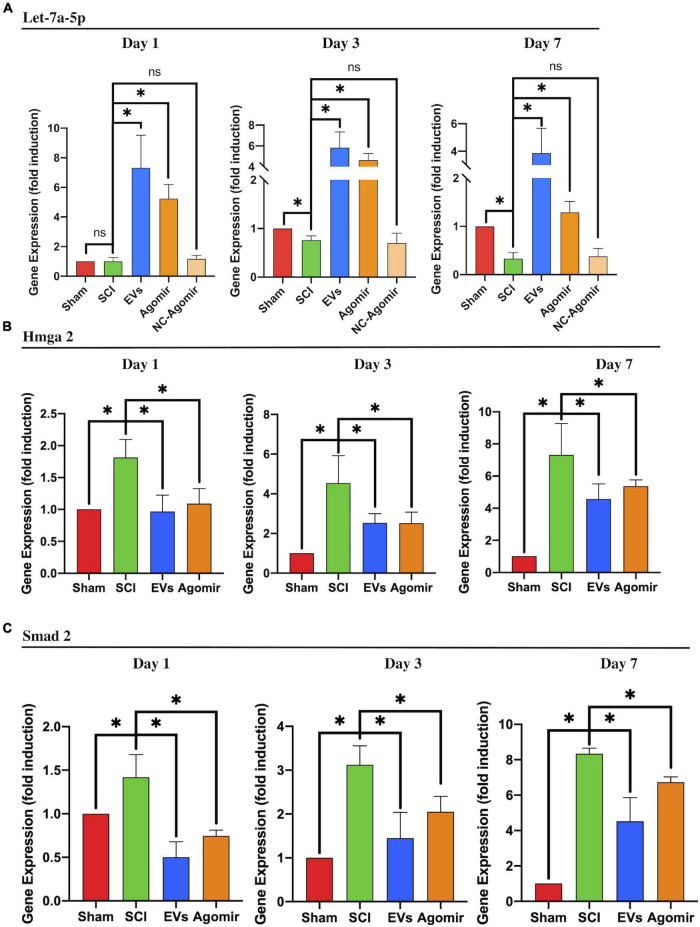
BMSC-EVs and let-7a-5p agomirs inhibited the expression of SMAD2 by targeting HMGA2. **(A)** The injection of BMSC-EVs increased let-7a-5p expression in injured lesion at different time points following SCI. Similarly, the let-7a-5p agomirs increased let-7a-5p expression in the early time points following SCI (*n* = 3). **(B,C)** In SCI rats, the expression of HMGA2 and SMAD2 in the injured lesion was markedly upregulated compared with the sham rats. Both BMSC-EVs or let-7a-5p agomirs were able to reduce HMGA2 and SMAD2 expression in SCI rats (*n* = 3; data are mean ± S.D; Student’s *t-*test was used for comparisons; gene expressions were normalized to GAPDH (for mRNA) and U6 snRNA (for miRNA). *p < 0.05. The term BMSC-EVs is abbreviated as EVs and let-7a-5p agomir is abbreviated as agomir.

*In vivo*, HMGA2 and SMAD2 expression was analyzed in the injured lesion sites from SCI, BMSC-EV-treated, and let-7a-5p agomir-treated rats, respectively. In SCI rats, the expression of let-7a-5p was significantly reduced at days 3 and 7 post-injury (Figure 5A) compared with the sham rats. In contrast, let-7a-5p expression was markedly increased after treatment with BMSC-EVs (Figure 5A). In accordance with the alternation of let-7a-5p expression, the expressions of HMGA2 and SMAD2 was upregulated in the injured lesion sites following SCI (Figures 5B,C). Moreover, the injection of BMSC-EVS or let-7a-5p markedly reduced the expression of HMGA2 and SMAD2 (Figures 5B,C). This indicated that BMSC-EVS was able to upregulate the expression of let-7a-5p, which, in turn, repressed HMGA2 and SMAD2 expression in the injured lesion sites following SCI.

### The Transfection of let-7a-5p Inhibitors or Antagomirs Abolished the BMSC-Extracellular Vesicle-Induced Regulation of the HMGA2/SMAD2 Axis

**FIGURE 6:**
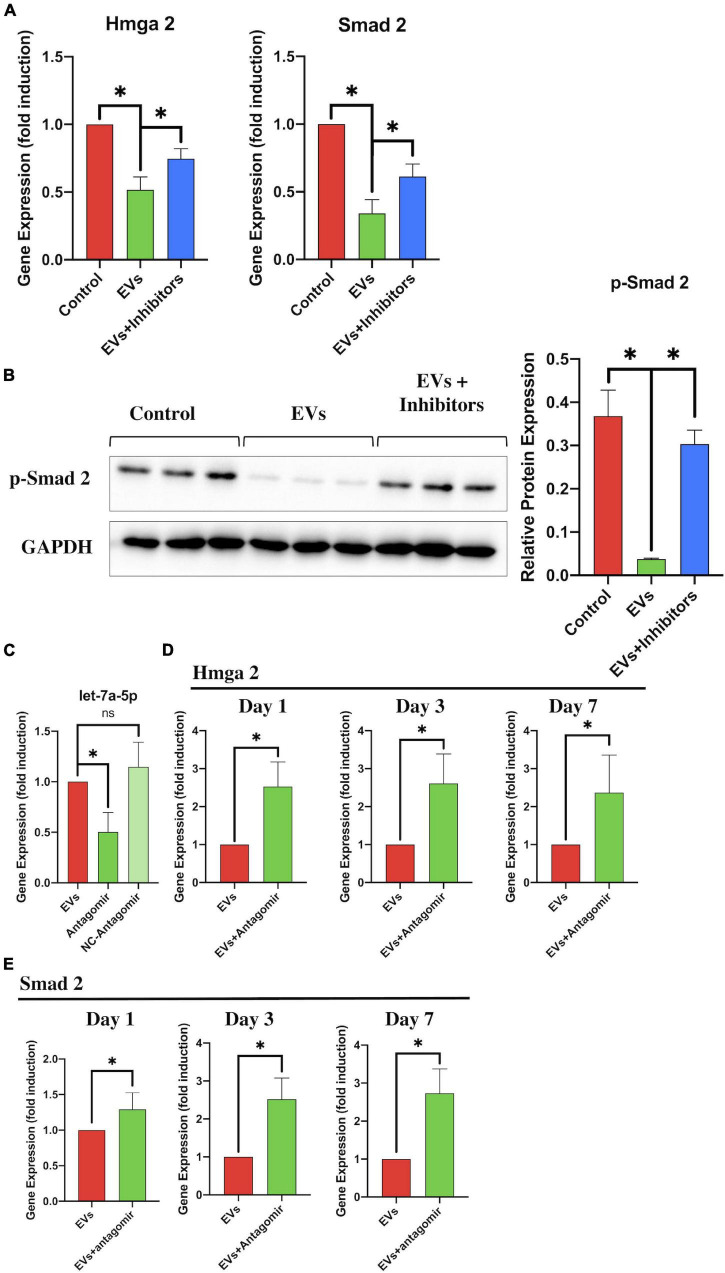
let-7a-5p inhibitors repressed the BMSC-EV-induced effects on the HMGA2/SMAD2 axis. **(A)** RT-PCR analysis revealed that the expression of HMGA2 and SMAD2 in NSCs was reduced by the addition of let-7a-5p inhibitors in the presence of BMSC-EVs (*n* = 3). **(B)** Western blot analysis also revealed that BMSC-EVs were able to reduce the expression of p-SMAD2 and that this BMSC-EV-induced regulation was partly attenuated by the addition of let-7a-5p antagomirs after 3 days of NSC culturing. **(C)** PCR analysis confirmed that the let-7a-5p antagomirs could reduce let-7a-5p expression (*n* = 3). **(D,E)** Compared with the BMSC-EV-treated rats, the let-7a-5p antagomir injection repressed the BMSC-EV-associated downregulation of HMGA2 and SMAD2 expression in the early phase of SCI, leading to an increase in HMGA2 and SMAD2 expression in the injured lesion (*n* = 3; data are shown as mean ± S.D; Student’s *t-*test was used for comparisons). Gene expressions were normalized to the GAPDH (for mRNA) and U6 snRNA (for miRNA). *p < 0.05. The term BMSC-EVs is abbreviated as EVs and let-7a-5p agomir is abbreviated as agomir.

To further identify that the BMSC-EV-regulated effects on the HMGA2/SMAD2 axis were associated with let-7a-5p within the EVs, we transfected the let-7a-5p inhibitors into NSCs in the presence of BMSC-EVs and detected the expression of HMGA2 and SMAD2 after 3 days of culture. Compared with the control groups, the addition of BMSC-EVs markedly reduced the expression of HMGA2 and SMAD2 in NSCs (Figure 6A). Moreover, the BMSC-EV-induced downregulation of HMGA2 and SMAD2 expression was partly abolished by transfection with let-7a-5p inhibitors (Figure 6A). These results were consistent with the western blot analysis, showing that the transfection of let-7a-5p inhibitors increased p-SMAD2 expression in the presence of BMSC-EVs (Figure 6B). In addition, we detected HMGA2 and SMAD2 in SCI rats that received BMSC-EV injection with or without let-7a-5p antagomirs. This revealed that the BMSC-EV-induced downregulation of HMGA2 and SMAD2 expression was partly attenuated by the addition of let-7a-5p antagomirs (Figures 6C,D,E). These data suggest that the BMSC-EV-related regulation of HMGA2 and SMAD2 expression was partly associated with let-7a-5p.

## Discussion

Mesenchymal stem cells are one of the most widely used cell types in cell-based clinical trials for neurological impairments such as spinal cord injury, stroke, Alzheimer’s disease, and neurodegenerative diseases (Karussis et al., 2008; Assinck et al., 2017; Dabrowska et al., 2019; Staff et al., 2019). They exert beneficial biological effects largely *via* the following: mediating the inflammatory process by limiting the pro-inflammatory cytokines and increasing the anti-inflammatory cytokines; protecting the surviving nerve cells from apoptosis; providing various neurotrophies to promote the regrowth of neurons, accelerating the remyelination of axons, and preventing the over-formation of glial scars. Moreover, a large number of studies have reported that most of these biological effects of MSCs contribute to MSC-secreted EVs, which enrich various proteins, lipids, and miRNAs (Phinney et al., 2015; Keshtkar et al., 2018; Harrell et al., 2019). Therefore, MSC-EV-based therapies have been used in many different animal models and have achieved promising outcomes. In spinal cord injury, a recent study by Li et al. (2020) revealed that mobilization of MSC-EVs in an adhesive hydrogel markedly improved neurological recovery by promoting neuronal regrowth and limiting secondary damage. Consistent with the present study, treatment with MSC-EVs significantly increased the regrowth of neurons and their outgrowth in the astrocytic scar boundary around the cavity.

Although the biological effects of MSC-EVs have been confirmed to be beneficial, the miRNAs and their mechanisms responsible for MSC-EV-induced effects have not been identified. Among all the miRNAs secreted from BMSCs, let-7 is the first known human miRNA, identified in *Caenorhabditis elegans* (Pasquinelli et al., 2000; Reinhart et al., 2000). It is abundantly expressed in the central nervous system (CNS) and has been reported to contribute to the development of the CNS and neurogliogenic decisions (Pena et al., 2009; Rehfeld et al., 2015; Xia and Ahmad, 2016). For example, in a neural development model, Balzer et al. (2010) found that during neurogenesis, Lin 28 was able to influence cell fate by regulating let-7. Further study by Patterson revealed that let-7 itself was able to switch the differentiation of neural progenitors from glial lineages directly toward neuronal lineages (Patterson et al., 2014). In the present study, it was revealed that *in vitro*, the addition of let-7a-5p mimics to NSCS increased the percentage of neurons. *In vivo*, the treatment of let-7a-5p could promote the outgrow of neurite into the astrocytic scar. These results, consistent with previous studies, indicated that the let-7a-5p was able to promote the differentiation of NSCs into neurons.

TGF-β plays a critical role in the regulation of a wide array of biological processes in various systems, including the CNS (Luo, 2017; Miyazawa and Miyazono, 2017; Stewart et al., 2018). The activation of TGF-β phosphorylates its downstream protein SMAD2, which in turn forms a complex with the co-Smad and translocases to the nucleus, where the complex exerts its function *via* the activation or repression of gene expression (Han et al., 2021). TGF-β has been reported to have a closely relationship with the balance between astrocytes and oligodendrocytes, the upregulation of TGF-beta signaling pathway could promote the differentiation of oligodendrocyte progenitors toward astrocytes (Zhang et al., 2010). Moreover, recent studies have shown that the activation of TGF-β also contributes to the upregulation of chondroitin sulfate proteoglycans (CSPGs) (Susarla et al., 2011; Jahan and Hannila, 2015), which are known as axon inhibitors. The overexpression of CSPGs in spinal cord lesions, especially in astrocytic scars, could act as barriers to stop the neurite outgrow or axons growth through the astrocytic scars, resulting in the neuroplasticity failure following SCI (Tran et al., 2018). Therefore, the inhibition of the TGF-β/Smad signaling pathway is considered a promising therapy to promote neurite outgrowth of neurons and avoid the over-formation of astrocytic scars.

In addition, HMGA2 has been reported to be an enhanced regulator that promotes TGF-beta/Smad signaling. A study by Xia and Ahmad (2016) revealed that let-7a could affect the generation of neurospheres and their differentiation likely by inhibiting HMGA2 expression. They also revealed that the let-7a-induced effects were abolished in the presence of ΔHMGA2. Similarly, in the present study, we found that treatment with let-7a-5p could inhibit the expression of HMGA2, inducing downregulation of SMAD2 expression. Taken together, let-7a-5p was able to promote the differentiation of NSCs into neurons and the regrowth of neurons and their outgrowth of neurites in SCI rats by targeting HMGA2.

Nevertheless, TGF-β has also been shown to be an anti-inflammatory cytokine. It could directly repress the upregulation of pro-inflammatory cytokines (Rustenhoven et al., 2016) or attenuate inflammation through inhibition of the NF-κB pathway (Miyazawa and Miyazono, 2017). Studies by Noh et al. (2016) and Xu et al. (2020) have pointed out that MSCs can regulate the properties of microglia and suppress neuroinflammation *via* TGF-β secretion. In a recent study (Han et al., 2021), we found that blocking TGF-β signaling in different phases of SCI exerts a distinct outcome. In the early phase of SCI, blocking TGF-β signaling might attenuate the anti-inflammatory process causing the spread of destructive inflammation, resulting in increased apoptosis or death of the adjacent nerve cells in the lesion sites. Therefore, in the acute phase of SCI, TGF-β is considered to be a beneficial anti-inflammatory cytokine that limits inflammation following SCI. In the present study, although the addition of let-7a-5p reduced SMAD2 expression in the early stage of SCI post-injury, the spread of the destructive inflammation was not noted. This could be because let-7a-5p only partially repressed the activation of SMAD2 and did not hinder the TGF-β’s downstream signaling pathways or the crosstalk with other signaling pathways (Luo, 2017). Moreover, it has been reported that let-7a itself had anti-inflammatory effects, which might compensated for the loss of TGF-β-induced anti-inflammatory effects (Li et al., 2019; Sui et al., 2019; Zhang et al., 2019). However, in the present study, we did not have direct evidence to prove whether let-7a-5p could increase the nerve cells survival by mediating the inflammation following SCI.

In addition, evidence from the present study indicated that the BMSC-EV-induced effects on the mediation of NSC differentiation were partly dependent on the secretion of let-7a-5p. For example, BMSC-EVs and let-7a-5p had similar effects on mediating the differentiation of NSCs, both of which were able to promote the differentiation of NSCs into neurons and increase the amount of neurons and neuron outgrowths in SCI rats. Likewise, the mechanisms of BMSC-EVs and let-7a-5p are similar, both reducing SMAD2 expression by targeting HMGA2; therefore, inhibiting let-7a-5p function blocked the effects of BMSC-EVs. *In vitro*, the BMSC-EV-induced effects on NSC differentiation were partly abolished by the addition of let-7a-5p inhibitors, resulting in a reduction in the percentage of neurons. *In vivo*, co-treatment with BMSC-EVs and let-7a-5p antagomirs inhibited neurite outgrowth in astrocytic scars compared with rats that received only BMSC-EVs. When attempting to study the mechanisms involved, HMGA2 and SMAD2 expression was notably decreased by treatment with BMSC-EVs *in vitro* and *in vivo*. This BMSC-EV-associated downregulation of HMGA2 and SMAD2 was abolished by the addition of let-7a-5p inhibitors/antagomirs. These results indicated that the effects of BMSC-EVs were associated with the secretion of let-7a-5p.

Another notable result is that although the BMSC-EV-induced regrowth of neurons in SCI rats was markedly repressed by the addition of let-7-5p antagomir, no significant difference of the neurological outcome was noted in these two groups. It could be explained by two reasons: firstly, let-7a-5p was one of the miRNAs secreted by BMSCs, some other miRNAs or proteins were also reported to be include in the BMSC-EVs and to be related with the mediation of the differentiation of NSCs (Song et al., 2020; Han et al., 2021; Zhang et al., 2021). Therefore, although the effects of let-7a-5p was abolished, the other miRNAs or proteins within BMSC-EVs might exert their effect to partly compensate for the let-7a-5p-induced effects. Secondly, the ability of the reorganization of the neural circuit was also closely associated with the neurological recovery following SCI (Freund et al., 2019). The appropriate reorganization could promote the recovery of motor or sensation, but inappropriate reorganization might lead to the neuropathic pain or spasm, which even worsens the neurological outcomes (Murray and Goldberger, 1974; Tan and Waxman, 2012; Tan et al., 2012). It explained that in some SCI rats, although the histology of injured lesions was similar, the neurological outcome might be distinct.

In conclusion, BMSC-EV-secreted let-7a-5p mediates the differentiation of NSCs and promotes the regeneration of neurons and their neurite outgrowth in glial scars by targeting HMGA2.

## Data Availability Statement

The raw data supporting the conclusions of this article will be made available by the authors, without undue reservation.

## Ethics Statement

The animal study was reviewed and approved by the Ethics Committee of Anhui Medical University.

## Author Contributions

CS and YW contributed to the research design. YW contributed to the manuscript writing. CS contributed to the manuscript editing. TH and PS contributed to the injury testing on the rats, tissue processing, and immunohistochemistry. ZW and RG contributed to the cell culturing, Western-blot, and ELISA arrays. YL and JA contributed to the RT-PCR analysis. All authors read and approved the final manuscript.

## Conflict of Interest

The authors declare that the research was conducted in the absence of any commercial or financial relationships that could be construed as a potential conflict of interest.

## Publisher’s Note

All claims expressed in this article are solely those of the authors and do not necessarily represent those of their affiliated organizations, or those of the publisher, the editors and the reviewers. Any product that may be evaluated in this article, or claim that may be made by its manufacturer, is not guaranteed or endorsed by the publisher.
