# Supplementary material for: Micro-RNA let-7a-5p Derived From Mesenchymal Stem Cell-Derived Extracellular Vesicles Promotes the Regrowth of Neurons in Spinal-Cord-Injured Rats by Targeting the HMGA2/SMAD2 Axis
Source: Front Mol Neurosci. 2022 Mar 25;15:850364. doi: 10.3389/fnmol.2022.850364 (PMC8990843; doi:10.3389/fnmol.2022.850364)

**Supplementary Figure 1**

**A.** Identification of BMSC-EVs by transmission electron microscopy. **B.** Detection of the diameter of BMSC- EVs by dynamic light scattering. **C.** Analysis of CD9, CD63, TSG101, and CD 90 expression by western blot


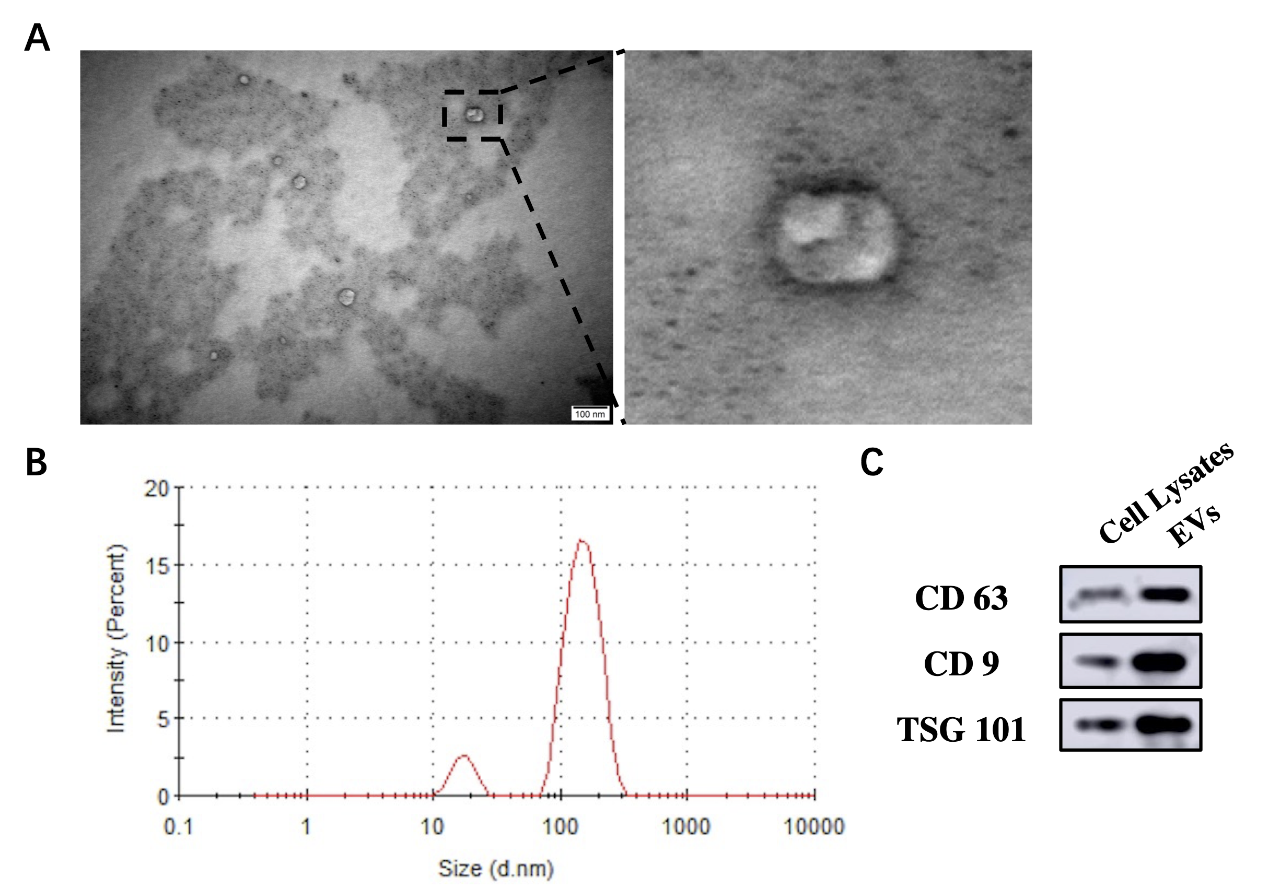

Supplement: Supplementary file 1 [file Data_Sheet_1.docx]
